# Clinical Endocrinology—Time for a Reset?

**DOI:** 10.1210/jendso/bvae024

**Published:** 2024-02-12

**Authors:** Stafford L Lightman

**Affiliations:** Translational Health Sciences, The Medical School, University of Bristol, Dorothy Hodgkin Building, Whitson Street, Bristol BS1 3NY, UK

**Keywords:** glucocorticoids, rhythms, Addison’s, Cushing’s, hyperaldosteronism, stress

## Abstract

Measurement of blood levels of circulating hormones has always been the cornerstone of the biochemical diagnosis of endocrine diseases, with the objective of detecting hormone excess or insufficiency. Unfortunately, the dynamic nature of hormone secretion means single-point measurements of many hormones often lack diagnostic validity. Endocrinologists have devised complex dynamic tests as indirect assessments of the functioning of the hormone system under investigation. Recent advances in the measurement of dynamic hormone changes across the day now offer an opportunity to reconsider whether there might be better ways both to diagnose and to monitor the therapy of endocrine conditions.

What is the role of the clinical endocrinologist? I should like to propose that our role should be to help provide the optimal pattern of hormone levels in the body to ensure physical and mental well-being. If we accept this premise, then we will also need:

1. To understand the normal patterns of hormones across the day in males and females, young and old, with different body mass indexes, across the menstrual cycle and in response to both internal stressors such as disease and inflammation and external stressors such as exercise and chronodisruption.
2. To be able to diagnose abnormal patterns of hormone secretion.
3. To be able to provide treatment that allows the return of normal patterns of hormones.

How far have we come in achieving these aims? During the late 19th and early 20th centuries, we recognized that, in addition to disorders of the gonads, certain clinical conditions—such as hypothyroidism, myxoedema, Grave’s disease, Diabetes mellitus, Addison’s disease, Cushing’s disease, acromegaly, adrenal and extra-adrenal phaeochromocytoma, and parathyroid tetany were associated with the malfunction of hormone-secreting glands [1]. This was followed by a massive increase in our understanding of the networks regulating hormone secretion and the mechanisms of hormone signaling and a realization that feedforward activation of endocrine glands and the feedback regulation by hormones (or the substrates they regulate) to control their own secretion provided a framework that was disrupted in endocrine diseases. This allowed endocrinologists to design diagnostic strategies to assess whether, in any 1 individual, their hormonal system was either overproducing hormones or underproducing them. The classic diagnostic tenet for endocrine diagnosis became: if we suspect an underproduction of hormone, try to stimulate the relevant gland, and if we suspect overproduction, try to inhibit it.

To a totally naïve onlooker, this seems rather illogical. Surely if we suspect over- or underproduction of a hormone or hormones, what we need to do is to measure them and assess whether they are over- or undersecreted. The problem, of course, is that most hormones are extremely dynamic, not only having their own circadian and ultradian oscillations but also varying across the menstrual cycle and in response to multiple daily stimuli from the everyday hassles of life to food intake and changes in sleep patterns [2]. The only way to take these into account would be to perform 24-hour blood sampling, but even if this were practical, it would not be successful as (1) it would need admission to a very expensive clinical investigation unit, (2) sleep would be disturbed in this new artificial environment, and (3) the whole process can be stressful and thus alter the endocrine output. In the light of these problems, clinical endocrinologists have shied away from determining whether patients have normal patterns of hormone secretion. The one exception has been the use of saliva as a surrogate of blood hormone levels. The measurement of salivary cortisol/cortisone changes across the day has achieved some clinical validity, and late-night measurement has been shown to be a valuable screen for ACTH-dependent hypercortisolism [3]. Unfortunately, however, continued sampling once the patient is asleep is not possible, and the majority of patients with a ≥1 elevated late-night salivary cortisol or cortisone result did not have Cushing’s syndrome, demonstrating that a single time point elevated level has poor specificity and predictive value.

Technology has now moved on, most particularly via dramatic developments in wearable sensor technology. The potential power of these sensors is clearly illustrated by the revolution taking place in diabetes mellitus, where the use of continuous glucose monitors can provide a high level of personalized precision medicine advancing the single hemoglobin A1c determination to complex glucose profiles with analysis of “time in range.” A sensor device for insulin measurements would further improve the indices for exogenous insulin administration [4]. This knowledge of dynamic changes in glucose across the day can not only improve blood sugar control but also empower patients to understand and improve their disease management.

What are the potential values and implications of this sensor revolution for other endocrine diseases?

1. The first lesson is that if we have wearable sensor technology, we should be able to monitor a patient's hormone levels in their own home/work environment—which is, of course, what we actually want to know.
2. Since most of these new technologies are measuring hormones in interstitial fluid, we are measuring the free unbound hormones in the same tissue environment that provides hormonal access to cell membrane or intracellular receptors. This environment will also be responsive to local regulatory mechanisms such as deiodination and activation of thyroid hormones and the regulation of glucocorticoids by 11βHSD1 and 2.
3. This technology will allow us to ascertain the full pattern of hormone secretion across the day, including both the circadian and ultradian rhythms, response to everyday hassles, and, most importantly of all, the levels during the hours of sleep that are so important for many regulatory processes. Obvious applications include (1) improved understanding of which patients with adrenal incidentalomas actually have abnormal cortisol (or aldosterone) secretion over the full 24 hours, providing new data to update practical guidelines on management [5]; (2) define which patients with a history of glucocorticoid-induced adrenal insufficiency still need to have continued replacement therapy [6], and (3) not only improving and simplifying criteria for the diagnosis of Cushing’s disease but, more important, in terms of epidemiology, improving and simplifying our diagnosis of primary hyperaldosteronism in hypertension. As John Funder points out [7], despite the fact that at least 30% of hypertensives who are appropriately screened have primary hyperaldosteronism—and this group has a 3-fold higher cardiovascular risk profile than essential hypertensives with the same blood pressure—less than 1% of hypertensives are screened for primary hyperaldosteronism. Surely we can do better, and hopefully wearable technology will allow easier diagnostic pathways for these patients.

The importance of being able to measure hormone levels across the day is also important in areas of therapeutics, including hormone replacement, for instance in adrenal insufficiency and congenital adrenal hyperplasia (CAH). It is often poorly appreciated that patients show major interindividual differences that are not picked up by our current monitoring tests. This is well illustrated in Fig. 1, which shows that 1 subject—despite a flat synacthen test—has an endogenous surge of cortisol in anticipation of awakening whereas another does not! It is often a mystery why some patients on glucocorticoid replacement feel awful when they wake up or indeed at other times of the day, while others seem fine, despite similar replacement regimens. We should now be able to personalize replacement to ensure optimal cover across the full 24 hours. We should also be able to characterize the normal physiological responses to the “stress” of surgery and thus improve our replacement for patients with adrenal insufficiency. Furthermore, since no blood is needed for interstitial fluid monitoring of hormone levels, this technology is also suitable for repeated estimation of hormone levels in children and infants [8] (Fig. 1).

**Figure 1.**
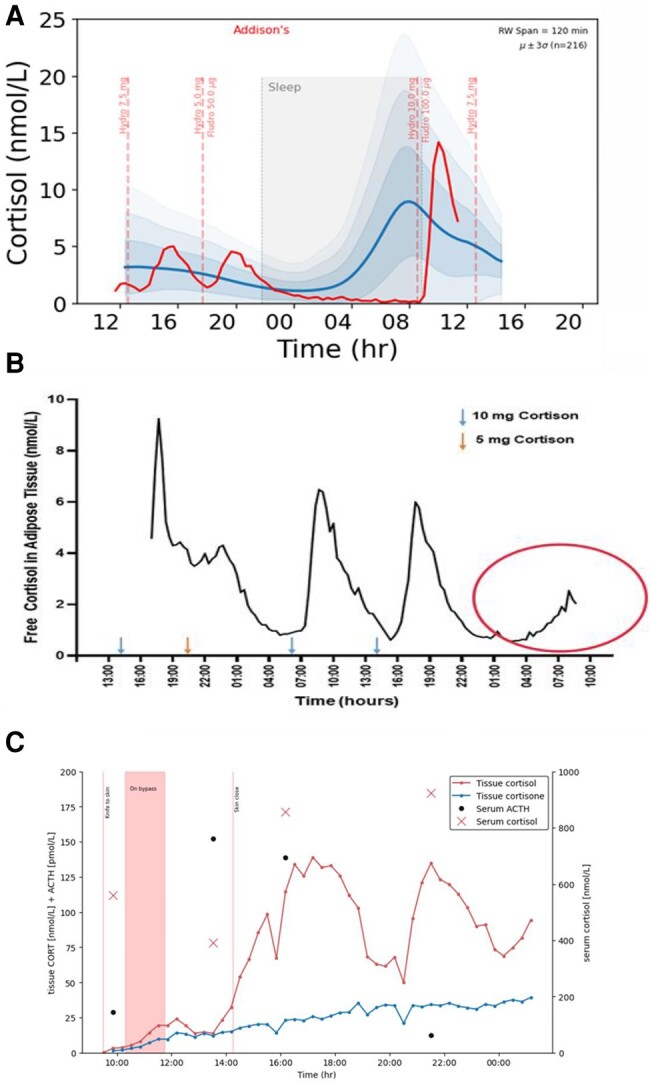
Panel (A) (from the Ultradian Consortium, see [9]), and panel (B) (unpublished data Saevik and Husebye) demonstrate the daily profile of 2 individuals with long-standing Addison’s disease on hydrocortisone (A) (compared with data from 214 normal controls [9]) or cortisone acetate (B) replacement. Note the interindividual differences in morning cortisol—The subject in (A) has severe morning hypocortisolaemia, while, despite a flat synacthen test, the patient in (B) shows an endogenous morning increase in cortisol circled in red. Panel (C) (unpublished data from Fudulu et al see [8]) shows the dynamic changes in cortisol and cortisone during and following open heart surgery for a Glenn shunt in an infant of 1 year 2 months. Note the delayed hypothalamic-pituitary-adrenal activation presumably related to the inflammatory response to sternotomy rather than the operative process itself.

How do we now proceed to make use of these new technologies? The first action must be to establish a dynamic normal range across the day—and achieve a gold standard that can be used as the basis for future diagnostics and for the validation of other sensing modalities. This has now been achieved for adrenal corticosteroid secretion using the technique of ambulatory microdialysis combined with ultrasensitive liquid chromatography-tandem mass spectrometry [9]. The next step must be the development and validation of algorithms and key dynamic biomarkers for the diagnosis of specific diseases such as Cushing’s disease and primary hyperaldosteronism and the improvement in hormone replacement in patient groups including those with adrenal insufficiency, CAH, hypothyroidism, and hypogonadism. This information about dynamic changes in corticosteroids across the day will also allow a more appropriate way to investigate the role of the hypothalamic-pituitary-adrenal axis dysfunction in stress related disorders such as posttraumatic stress disorder, depression, and autoimmune disease.

Once the key diagnostic biomarkers have been defined, we will also be able to refine which data are most important for our diagnostic tests. We should, for instance, be able to sample at much more targeted times rather than the full 24 hours, thus reducing the cost of the highly sensitive mass spectrometry. The availability and cost of analyses will, however, need to respond to market forces before this technique can come into routine clinical use.

In addition to systems that obtain interstitial fluid analytes for subsequent assay, there has also been increasing interest in the development of remote sensors to measure hair, sweat, and interstitial fluid hormone levels using novel bioanalytical platforms [10-23]. Biosensing with antibodies/aptamers, quantum dots, or molecularly imprinted polymers in combination with microfluidic devices and nanoelectronics offer the potential for low-volume samples and signal detection suitable for scalable production and multiplexing. Although there are many problems that will need to be solved before any of these techniques reach the validity achieved by microdialysis, there is every reason to believe that multiplexed sensors will eventually reduce costs and provide a high level of personalized endocrine care.

It is important that we do not only look at this changing scene in endocrine practice from the view of the medical practitioner. Patients are also very keen to gain a better understanding of their medical condition, and together with their support groups are voicing increasing requests to be actively involved in their own care. One major aspect of this is to share an improved understanding of how their hormone levels (cortisol/thyroid hormones/reproductive hormones, etc) across the day correlate with their symptoms. The ability to discuss their pattern of hormones across the day with their endocrinologist will not only help empower patients but also improve the knowledge base for discussions with their medical professionals on how to improve their therapy. A good example can be seen in the unexpected endogenous secretion of cortisol in one patient but not in another shown in Fig. 1, demonstrating how these techniques will reveal not only the (currently undiagnosed) great degree of variation between normal subjects but also practical information such as whether some patients with residual adrenal function may have a clinically important preawakening increase in cortisol whereas other subjects will have overnight hypocortisolaemia.

Diagnosing abnormalities of hormone secretion is, of course, only the first part of the role of the clinical endocrinologist. The second part is the provision of treatment that allows the return of normal patterns of hormones. This is a subject worthy of its own perspective article, but suffice it to say this will only be possible if we first of all understand what is normal. Preliminary studies, however, do suggest that a truly physiological pattern of cortisol replacement may be very important in at least some patients [24, 25].

Are we saying goodbye to our much loved—and hated—dynamic function tests? Not quite yet. First, we will need new clinical data to redefine who does and does not need surgical or medical treatment. This data, however, will provide us with well-validated algorithms for what is normal for people in their own home environments and become a secure basis for assessing whether someone’s hormone replacement protocol really is optimal, whether it is sufficient to inhibit a morning rise of androgens in CAH, or whether it indicates the need for removal of an incidentaloma, etc.

Clearly, we are only at an early stage in moving toward these new technologies, but cost and patient pressure will undoubtedly move us in this direction. Validation at each step will be key—but with the appropriate sensors, there is no reason we should not be able to diagnose and manage many areas of adrenal, thyroid, reproductive, pituitary, and metabolic disease with home-based hormone measurement. This will be an exciting decade.

## Data Availability

Data sharing is not applicable to this article as no datasets were generated or analyzed during the current study.
